# Health Related Quality of Life among schoolchildren aged 12–13 years in relation to food hypersensitivity phenotypes: a population-based study

**DOI:** 10.1186/s13601-017-0156-9

**Published:** 2017-07-03

**Authors:** Åsa Strinnholm, Linnéa Hedman, Anna Winberg, Sven-Arne Jansson, Viveca Lindh, Eva Rönmark

**Affiliations:** 1 Department of Public Health and Clinical Medicine, Occupational and Environmental Medicine, OLIN Unit, Umeå University, 901 87 Umeå, Sweden; 2 Division of Nursing, Department of Health Sciences, Luleå University of Technology, 971 87 Luleå, Sweden; 3 Department of Clinical Sciences, Pediatrics, Umeå University, 901 87 Umeå, Sweden; 4 Department of Nursing, Umeå University, 901 87 Umeå, Sweden

**Keywords:** Adolescents, Food hypersensitivity, Health Related Quality of Life, Phenotypes of food hypersensitivity, Population-based cohort study

## Abstract

**Background:**

While Health Related Quality of Life has been investigated among children with IgE-mediated food allergy, less is known about quality of life among children with other types of hypersensitivity to food. The aim of this study was to investigate Health Related Quality of Life (HRQL) in children with and without food hypersensitivity. Further, we compared HRQL between children with different phenotypes of food hypersensitivity.

**Methods:**

In a large population-based cohort of schoolchildren in Northern Sweden, the parents of 2612 (96% of invited) completed a questionnaire. All 125 (5%) children who reported complete elimination of milk, egg, fish or wheat due to food hypersensitivity were invited to a clinical examination and 94 children participated. Of these, 75 children also completed a generic (KIDSCREEN-52) and a disease-specific HRQL questionnaire (FAQLQ-TF). Thereafter, these children were categorised into the different phenotypes: current food allergy, outgrown food allergy, and lactose intolerance. Additionally, 209 children with unrestricted diets answered the generic questionnaire.

**Results:**

The median score of all KIDSCREEN-52 domains were above the population norm of 50 both in children with and without food hypersensitivity. No significant differences in distribution in generic or disease-specific HRQL were found between children with or without food hypersensitivity. There were no significant differences in HRQL between children with different phenotypes of food hypersensitivity. However, children with current food allergy tended to have the lowest HRQL. Further, poor HRQL defined as ≥75th percentile for the disease specific score was significantly more common in the current food allergy phenotype in the domain Emotional impact and the total FAQLQ, compared to the other phenotypes.

**Conclusions:**

In this population-based study, 12–13 year old children reported good HRQL regardless of having food hypersensitivity or not. However, the children with the current phenotype reported lower HRQL than the other phenotypes.

**Electronic supplementary material:**

The online version of this article (doi:10.1186/s13601-017-0156-9) contains supplementary material, which is available to authorized users.

## Background

Reported food hypersensitivity (FHS) is common in the Western countries. However, there are large differences in prevalence between studies. In a meta-analysis investigating FHS in 51 studies, the prevalence of self-reported FHS varied from 3 to 35% due to differences in study methods, age of study populations and geographic settings [1]. The high and increasing prevalence of reported FHS [2, 3] may partly be attributed to an increasing awareness about FHS in the population, as well as public interest of different diets [4]. The term FHS includes food reactions of both immunological and non-immunological origins, and food allergy is a subgroup of FHS [5].

Elimination of foods due to FHS may negatively affect the Health Related Quality of Life (HRQL) [6]. HRQL can be defined as self-perceived health [7] since it is possible to have a chronic disease but still experience a good self-perceived HRQL. Since HRQL is individual and varies with age [6], it is suggested that when applicable, the questionnaires should be completed by the individuals themselves [8]. Usually, children are able to complete their HRQL questionnaire from the age of eight years, given that the questionnaires are age appropriate and that the child have reading skills [9]. Generic HRQL questionnaires are used for comparisons between different diseases, or between subjects with or without a disease [10]. Disease-specific questionnaires investigate HRQL related to a specific disease, e.g. food allergy [11].

The interest in HRQL in children and adolescents with food allergy has increased during the last decades, and a number of disease-specific HRQL questionnaires are now available for children [11, 12], adolescents [13, 14], and parents to children [15–18] with IgE mediated food allergy. However, there is a limited number of HRQL questionnaires available for the wider definition FHS [19, 20]. To our knowledge, no studies have explored HRQL among children with different phenotypes of FHS.

In a population-based cohort of schoolchildren, the paediatric cohort II within the Obstructive Lung Disease in Northern Sweden studies (OLIN), 5% of the children reported complete elimination of cow’s milk, hen’s egg, fish or wheat due to FHS at age 11–12 years [21]. The aim of the current study was to compare HRQL among children with and without complete elimination of cow’s milk, hen’s egg, fish or wheat due to FHS, and to study HRQL in relation to different FHS phenotypes. Since milk, egg, fish and wheat are staple foods in Western diet, we hypothesised that avoidance of these foods would have a negative impact on HRQL.

## Methods

### Study population

In 2006, all children in first and second grade (aged 7–8 years) in three municipalities in Northern Sweden were invited to a longitudinal study about asthma, rhinitis, eczema and FHS [22–24]. The current study was based on a study follow-up in 2010 when the parents of 2612 (96% of invited) children, now in ages 11–12 years, participated in a questionnaire survey [3]. Children in two of the municipalities were also invited to skin prick testing (SPT) with ten common airborne allergens, and 1657 (86% of invited) participated. The SPT were performed by a small group of well-trained research nurses.

### Parental questionnaire

The questionnaire included the International Study of Asthma and Allergies in Childhood questions [25] with added questions about symptoms and diagnosis of atopic diseases including FHS [3, 22]. The queries about FHS included; symptoms of FHS in relation to different foods, if elimination of the culprit food was partial or complete, and whether the child had a physician-diagnosis of celiac disease.

### Clinical examination

**Fig. 1:**
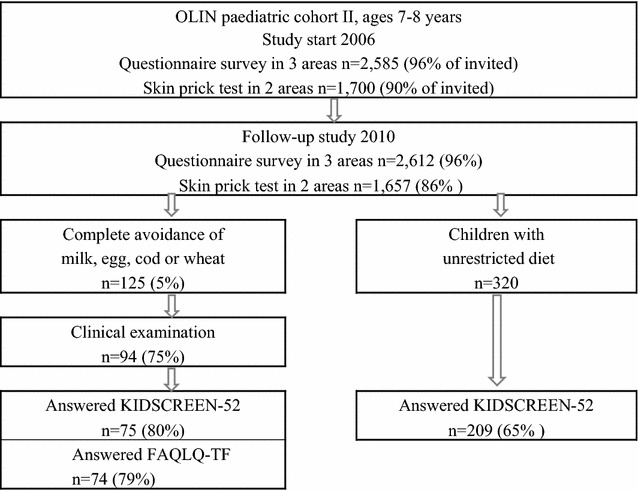
Study design and participation

Five percent (n = 125) of the children reported complete elimination of cow’s milk, hen’s egg, fish and/or wheat due to FHS [21]. During October and November 2010, these children were invited to a clinical examination including a structured interview, specific IgE to the culprit food and a celiac screen test (tissue transglutaminase IgA antibodies) and 94 children (75% of invited) participated [21]. The clinical examinations were performed by the same paediatric allergist (AW). At the time of the clinical examinations, 94 children, 75 (80% of participants) also answered a generic HRQL questionnaire and 74 children answered a disease-specific questionnaire. In addition, a random sample of 320 children without food hypersensitivity was invited to complete the generic HRQL questionnaire during the same period. The questionnaire was sent by mail to the children with unrestricted diet, and 209 (65% of invited) participated. The children were instructed to answer the questionnaire without interference of their parents. The study design and participation are presented in Fig. 1.

### Phenotypes of FHS

According to the results of the clinical examination, all children were subsequently categorised into the different FHS phenotypes. Among the 75 children who also answered the HRQL questionnaire the number of children in each phenotypes were: food allergy (n = 23), outgrown food allergy (n = 16) and lactose intolerance (n = 33). Three children could not be categorised to any of the FHS groups: one child was diagnosed as having celiac disease and two children declined participation in blood sampling. The categorisation was performed by the same paediatric allergist (AW) that performed the clinical examinations. The criteria used for the categorisation are presented in a previous study [21].

### The generic HRQL questionnaire

HRQL was measured using the Health-related Quality of Life Screening Instrument, KIDSCREEN-52, a generic self-report instrument for children and adolescents aged 8–18 years [10, 26]. The questionnaire has predominantly been used for studies in European countries [27]. The KIDSCREEN-52 measure HRQL in ten domains with underlying items: Physical Well-being (five items), Psychological Well-being (six items), Moods and Emotions (seven items), Self-Perception (five items), Autonomy (five items), Parent Relation and Home Life (six items), Financial Resources (three items), Social Support and Peers (six items), School Environment (six items) and Social Acceptance and Bullying (three items). The responses are given on a five-point scale where 1 is worst HRQL and 5 is the best HRQL. The computerised data was transformed into scales from 0 to 100 for each domain [26]. The instrument, including the Swedish version, has been previously validated [27]. The population norm is a mean of 50 and a standard deviation of ten assuming a normal distributed sample is included. A higher score indicate a better HRQL with 100 indicating the best quality of life and 0 the worst [10, 26].

### The disease-specific HRQL questionnaire

Since the children in our study were 12–13 years of age at the time they completed the questionnaires, we used the Food Allergy Quality of Life Questionnaire Teenager Form (FAQLQ-TF). The FAQLQ-TF questionnaire is a self-report instrument for measuring the impact of food allergy on the adolescent’s HRQL, at the ages 13–17 years [13]. The FAQLQ-TF questionnaires measure HRQL in three domains with underlying items: Allergen Avoidance & Dietary Restrictions (ten items), Risk of Accidental Exposure (six items) and Emotional Impact (seven items). The responses are given on a seven-point scale. The domain score is the sum of all question scores in the domain divided with number of completed questions in the domain. Total FAQLQ-TF score is the average score of all individual items and each score range from one (minimal impairment) to seven (maximal impairment). A higher score indicate a poorer HRQL. The questionnaire has been translated into Swedish, and used in a previous study [28] but is not yet validated in Sweden. The level of the clinical Minimal Important Difference (MID) is still undecided [29] though, a difference of 0.5–0.7 on a seven point scale has been suggested as clinically relevant [30, 31].

### Definitions

Any positive SPT: A positive reaction to at least one of the tested airborne allergens.

Asthma/rhinitis/eczema: Positive response to the question: “Has the child been diagnosed by a physican as having asthma/rhinitis/eczema?”

Food hypersensitivity (FHS): Reported complete elimination of cow’s milk, hen’s egg, fish and/or wheat due to food allergy or food hypersensitivity.

Heredity asthma/rhinitis/eczema: Reported parental history of asthma/rhinitis/eczema.

Heredity FHS: Reported parental history of food hypersensitivity.

### Statistical analyses

The statistical analyses were performed using the Statistical Package of Social Science Software version 22 (SPSS Inc. New York, USA). The Chi square test was used for comparison of categorical data. Since HRQL data was not normally distributed, the Mann–Whitney U test or the Kruskal–Wallis test was used to assess statistical significant differences between groups. A *p* value <0.05 was considered statistically significant. Poor HRQL was defined as ≥75th percentile for the FAQLQ-TF score and differences between groups were analyzed by Linear by linear association (Mantel–Haenszel).

## Results

### Basic characteristics of study participants

The prevalence of atopic diseases was significantly higher among children with FHS compared to children without FHS: asthma 25.3 versus 6.2%, rhinitis 28.0 versus 6.2% and eczema 32.0 versus 10.5%, as was family history of FHS, 50.7 versus 21.1% (p < 0.001 for all). There were no significant differences between children with and without FHS regarding allergic sensitization to airborne allergens, sex, living conditions, parental smoking or parental socio-economic status (Table 1).

**Table 1:** Comparison of atopic diseases and demographic factors between children with and without food hypersensitivity (FHS)

	FHS n = 75 % (n)	Non-FHS n = 209 % (n)	*p* value
Sex			
Girls	58.7 (44)	48.3 (101)	0.124
Boys	41.3 (31)	51.7 (108)	
Asthma	25.3 (19)	6.2 (13)	<0.001
Rhinitis	28.0 (21)	6.2 (13)	<0.001
Eczema	32.0 (24)	10.5 (22)	<0.001
Heredity FHS	50.7 (38)	21.1 (44)	<0.001
Any positive SPT^a^	46.3 (25)	33.0 (69)	0.069
*Living conditions*			
Current living			
House	86.1 (62)	79.6 (156)	
Apartment	13.6 (10)	20.4 (40)	0.108
Single parent household	8.0 (6)	10.2 (21)	0.581
Father smoke	13.5 (10)	13.7 (28)	0.967
Mother smoke	10.8 (8)	18.2 (37)	0.139
*Parental socioeconomic status*			
Professionals	33.2 (24)	24.4 (51)	
Self employed	4.0 (3)	6.2 (13)	
Intermediate non-manual	26.7 (20)	30.6 (64)	
Assistant non-manual	9.3 (7)	11.5 (24)	
Manual workers	14.7 (11)	11.5 (24)	
Manual workers service	8.0 (6)	10.0 (21)	
Unemployed	1.3 (1)	3.3 (7)	0.714

^a^Based on 54 children with food hypersensitivity and 209 children without food hypersensitivity who took part in skin prick testing

The responders and non-responders to the HRQL questionnaires did not differ regarding sex, atopic diseases, heredity of FHS, allergic sensitization or living conditions. Additional files 1 and 2 shows this in more detail.

### Generic HRQL among children with and without FHS

**Fig. 2:**
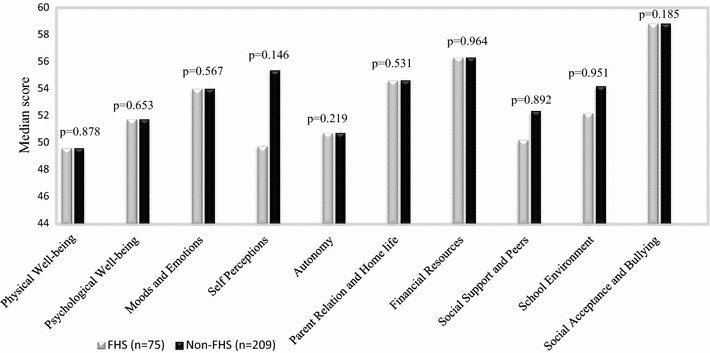
Median scores in KIDSCREEN-52 domains, among children with and without food hypersensitivity. A higher score indicate better HRQL. Differences between groups was analysed by Mann–Whitney U test

The KIDSCREEN-52 scores vary from 0 to 100, with 100 indicating the best quality of life and 0 the worst. The KIDSCREEN-52 domain specific median scores are presented in Fig. 2. The median value in all domains, among children with and without FHS were around 50 and above. No significant differences in distribution of HRQL were found when comparing children with or without FHS. In the Self-Perceptions domain, the median value tended to be lower among children with FHS compared to children without FHS, although the difference was not statistically significant.

Stratified analysis by sex were performed. Among girls, there was a significant difference between children with and without FHS in the distribution of HRQL in the domain Social Acceptance and Bullying (p = 0.037). In the other domains, no differences in distribution of HRQL were found, among neither girls nor boys (Table 2).

**Table 2:** Scores in KIDSCREEN-52 domains among girls and boys, with and without food hypersensitivity, respectively

KIDSCREEN-52 dimension	Girls			Boys		
	FHS (n = 44) Median (Q_1_–Q_3_) Mean	Non-FHS (n = 101) Median (Q_1_–Q_3_) Mean	p value*	FHS (n = 31) Median (Q_1_–Q_3_) Mean	Non-FHS (n = 108) Median (Q_1_–Q_3_) Mean	p value*
Physical well-being	49.6 (42.5–58.4)	49.6 (42.5–55.6)	0.641	49.6 (44.7–52.4)	49.6 (44.7–59.3)	0.521
	50.1	49.5		50.1	51.0	
Psychological well-being	51.8 (47.1–56.8)	51.7 (45.1–57.6)	0.447	51.8 (45.1–57.6)	54.5 (47.1–57.6)	0.172
	53.2	51.6		51.8	53.8	
Moods and emotions	50.2 (45.4–62.1)	54.0 (45.4–62.1)	0.702	54.0 (47.1–70.9)	55.7 (49.1–62.1)	0.982
	52.9	53.5		55.35	56.5	
Self perceptions	49.8 (46.1–56.6)	52.2 (45.3–60.1)	0.879	52.2 (49.8–69.8)	55.4 (52.2–60.1)	0.199
	52.7	52.8		55.8	57.6	
Autonomy	48.7 (45.2–60.5)	50.7 (47.8–56.3)	0.879	53.2 (46.8–60.5)	53.2 (48.7–60.5)	0.646
	51.8	53.6		52.8	54.6	
Parent relation and home life	54.6 (48.0–58.5)	54.6 (47.5–65.9)	0.691	54.6 (47.5–65.9)	54.6 (49.5–65.9)	0.759
	53.8	54.3		54.3	55.4	
Financial resources	56.3 (49.3–62.9)	56.3 (49.3–62.9)	0.945	56.3 (46.6–62.9)	56.3 (49.3–62.9)	0.942
	55.6	55.8		55.3	55.4	
Social support and peers	52.4 (48.3–58.1)	54.9 (47.1–58.1)	0.667	48.3 (45.1–58.1)	50.2 (45.1–58.1)	0.828
	53.8	54.1		51.5	51.8	
School environment	54.2 (50.4–61.9)	54.2 (48.6–61.9)	0.905	52.2(46.9–58.9)	52.2 (48.6–56.4)	0.660
	55.7	56.0		53.1	53.1	
Social acceptance and bullying	58.8 (48.1–58.8)	58.8 (58.8–58.8)	0.037	58.8 (48.1–58.8)	58.8 (48.1–58.8)	0.947
	52.6	55.7		52.6	52.6	

* The statistical significant differences between groups was measured by Mann–Whitney U test

### Generic HRQL in different FHS phenotypes

HRQL was compared between children with the different FHS phenotypes: current food allergy, outgrown food allergy and lactose intolerance. The KIDSCREEN-52 domain specific scores according to FHS phenotype are presented in Table 3. No significant differences in distribution were found between the phenotypes and the children without FHS. Children with current food allergy tended to have the lowest median values in several domains but the differences were not statistically significant.

**Table 3:** Scores in KIDSCREEN-52 domains among children with different food hypersensitivity phenotypes and children without food hypersensitivity

KIDSCREEN-52 dimension	Current food allergy n = 23 Median (Q1–Q3) Mean	Outgrown food allergy n = 16 Median (Q1–Q3) Mean	Lactose intolerance n = 33 Median (Q1–Q3) Mean	Non-FHS n = 209 Median (Q1–Q3) Mean	p value Across 4 groups*
Physical well-being	47.08 (42.53–55.60)	49.63 (40.97–63.06)	49.63 (44.73–55.60)	49.63 (43.08–55.60)	0.896
	48.87	51.46	50.49	50.29	
Psychological well-being	49.34 (45.10–54.49)	54.49 (44.21–68.49)	54.49 (47.12–68.49)	51.78 (47.12–57.60)	0.321
	49.22	53.98	54.79	52.79	
Moods and emotions	51.34 (42.50–62.06)	57.39 (47.64–70.90)	54.02 (47.15–62.06)	54.02 (47.15–62.06)	0.369
	49.94	57.24	55.65	55.07	
Self perceptions	49.76 (46.09–60.11)	52.18 (47.78–69.78)	50.97 (48.28–58.93)	55.38 (49.76–60.11)	0.593
	53.54	55.42	54.26	55.27	
Autonomy	48.69 (40.54–56.27)	53.33 (45.17–60.52)	53.22 (46.85–68.75)	50.77 (48.69–60.52)	0.211
	48.65	53.33	55.09	54.15	
Parent relation and home life	54.65 (47.50–60.36)	56.58 (46.17–65.87)	54.65 (47.50–58.53)	54.65 (47.50–65.87)	0.928
	53.25	55.03	54.33	54.88	
Financial resources	62.86 (46.59–62.87)	52.41 (47.26–62.86)	59.60 (53.39–62.86)	56.35 (49.27–62.86)	0.537
	55.74	53.93	56.75	55.55	
Social support and peers	48.35 (46.66–54.93)	49.30 (44.37–57.33)	54.93 (47.50–67.06)	52.38 (46.66–58.13)	0.463
	50.96	51.39	55.30	52.88	
School environment	52.23 (46.94–58.87)	56.55 (52.22–61.87)	52.22 (48.61–61.87)	54.22 (48.61–58.87)	0.758
	53.46	56.11	55.00	54.82	
Social acceptance and bullying	58.85 (42.19–58.85)	58.85 (48.07–58.85)	58.85 (48.07–58.85)	58.85 (48.07–58.85)	0.556
	50.93	53.22	53.99	54.12	

* The statistical significant differences between groups was measured by the Kruskal–Wallis test

### Disease specific HRQL in different FHS phenotypes

**Fig. 3:**
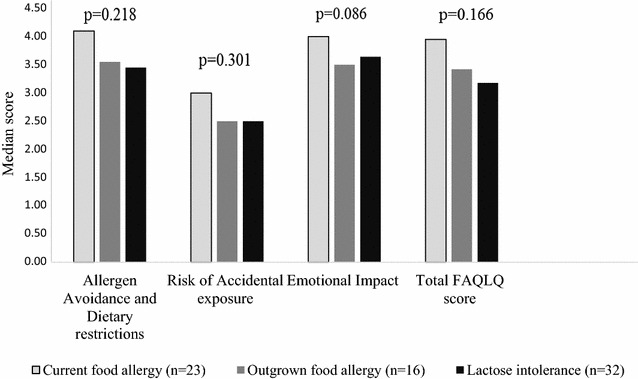
Median scores in FAQLQ-TF domains, by food hypersensitivity phenotypes. A higher score indicate a poorer HRQL. Differences between groups was analysed by the Kruskal–Wallis test. The score range of FAQLQ-TF is between 1 (best HRQL) and 7 (worst HRQL)

The FAQLQ-TF responses are given on a seven-point scale where 1 is the best HRQL and 7 is the worst HRQL. The FAQLQ-TF domain specific scores are presented in Fig. 3. No statistically significant differences in distribution of disease specific HRQL score were found between the different FHS phenotypes. However, the median score in all domains tended to be highest (indicating low HRQL) among children with *current food allergy* compared to *outgrown food allergy and lactose intolerance phenotypes*. Mean and median scores in FAQLQ-TF domains did not differ significantly by sex (Additional file 3).

**Fig. 4:**
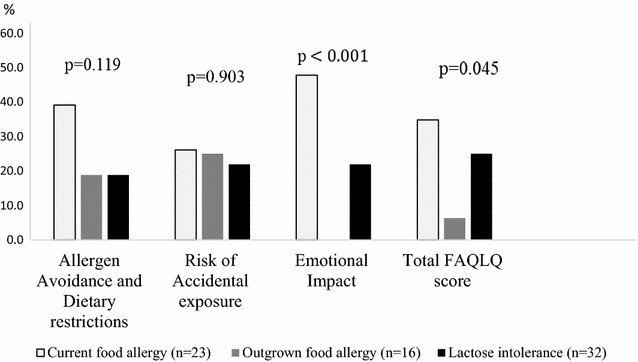
Prevalence (%) of poor HRQL (≥75 percentile) in FAQLQ-TF domains by food hypersensitivity phenotypes. A higher score indicate a poorer HRQL. Difference between groups was analysed by Linear by linear association (Mantel–Haenszel)

The proportions of poor HRQL, defined as ≥75th percentile are presented in Fig. 4. Poor HRQL was most common among children with *current food allergy*, and significantly so in the domain Total FAQLQ-score (p = 0.045) and Emotional Impact (p < 0.001).

## Discussion

According to European norms [10, 26], the children aged 12–13 years in this population-based study reported a good generic HRQL and there was no difference in HRQL between children with and without FHS. Further, no statistically significant differences in disease specific HRQL were found between children with the different FHS phenotypes: current food allergy, outgrown food allergy and lactose intolerance. However, the proportion of children having poor quality of life, defined as ≥75th percentile in the disease specific questionnaire, was more common in the current food allergy phenotype compared to the other phenotypes of FHS.

The participants in our study reported a good generic HRQL. Our result is in line with another Swedish population-based study comparing children aged eight years with or without atopic diseases including FHS. Overall, these children reported a good generic HRQL but children with atopic diseases, in particular asthma, had lower HRQL compared with children without atopic diseases [32]. Other factors than the disease itself may have an impact on the HRQL e.g. parental income [33], or being accepted by friends or not [34]. A possible explanation for the good HRQL among children with FHS in our study, and others as well, may be that FHS is common among children in Northern Europe [35]. Thus living with FHS or food allergy might be accepted as a normality and the restricted diet might therefore have a lesser impact on children’s daily life [36, 37].

The increased availability of milk and gluten free products in Sweden may affect the HRQL in a positive way e.g. it is easier to prepare meals for children with different types of FHS. On the other hand, it probably also increases the awareness of different types of diets. People may regard temporary or non-specific symptoms as symptoms of FHS leading to an un-necessary eliminated diet [4]. Interestingly, the participants in our study showed similar results compared to a Swedish study of randomly selected children aged 11–16 years, with the lowest score in the domain Physical wellbeing [38].

Country of origin may have an impact of HRQL. The Food Allergy Quality of Life Questionnaire-parent form was completed by parents of 1029 food allergic children from different parts of the US. The result was compared with 15 studies from different countries in Europe, using the same questionnaire. The HRQL was more impaired in the US population than the European populations [39]. Thus, it is important that translation and validation of HRQL questionnaires includes lingual as well as cultural translations, due to socioeconomic and cultural differences between countries [40].

To our knowledge, this is the first study investigating HRQL among different phenotypes of FHS in the general population. A number of hospital-based studies have described quality of life in children with IgE-mediated food allergy [13, 33, 41]. In hospital materials, poor HRQL has been related to severe symptoms to foods e.g. anaphylaxis, respiratory or cardiovascular symptoms [33, 41], multiple food allergies [11, 33, 41], comorbidity with atopic diseases [41] or allergy to specific foods e.g. cow’s milk and hen’s egg compared to allergy to peanuts or tree nuts [33]. In our population-based study, we did not find any statistical significant differences in HRQL between children with and without FHS, which includes a number of different adverse reactions to foods, including food allergy [5]. Food allergy is rarely associated with mortality [42] or daily physical food related symptoms, but the psychological stress of accidently being exposed to the culprit food can be a burden [6, 43]. Many children with FHS do not seek health care due to mild symptoms to foods [44]. Thus, it is likely that those who seek health care due to FHS have more severe symptoms and/or experience a higher impact on their daily life compared to those who do not [45]. This could explain the differing results in HRQL between hospital- based and our population-based study.

In analyses stratified by sex, we found no statistically significant differences in HRQL, except in the domain Social Acceptance and Bullying. In this domain, HRQL was more impaired among girls with FHS compared to girls without FHS, but this difference was not found among boys. It has been shown that adolescent girls generally experience a poorer HRQL compared to boys [46] which is in line with our results. Even though FHS is common among children, it may affect social life and create social exclusion [36] which may lead to bullying. In a previous study, children reported that they had been bullied because of their food allergy, mainly by classmates but also by teachers and school staff [34].

The clinical Minimal Important Difference of the disease specific FAQLQ is not yet decided [29]. We defined poor disease specific HRQL as ≥75th percentile and it was most common among children with current food allergy. A possible explanation is that severe symptoms are most common in this phenotype. While respiratory, cardiovascular and severe skin- and gastrointestinal symptoms are relatively common in food allergy [21, 41], lactose intolerance present with milder bowel symptoms [47]. Thus, children with current food allergy are probably more afraid of food reactions compared to children with other phenotypes of FHS associated with milder symptoms.

Interestingly, poor HRQL was also found among children with *outgrown food allergy*. This was however not surprising since these children had a convincing history of food allergy, but they still avoided the culprit food despite tolerance had been achieved [21]. It is well known that tolerance development is common in allergies to foods like egg and milk [48], but children may remain on an elimination diet even if tolerance is achieved [49]. Among our children with FHS, lactose intolerance was the most common phenotype, and these children also reported total elimination to cow’s milk [21]. Studies indicate that adolescents with lactose intolerance can drink smaller amounts of cow’s milk without symptoms [50]. Hence, after participating in our research program some of the children with FHS could reintroduce the eliminated food, partially or completely [21, 51] which may improve their HRQL. Reasons for staying on a restricted diet are fear of food reactions, difficulties to tolerate taste and textures of the eliminated food and unwillingness to change an approach to food that has become normality [51, 52]. Since reported FHS is common in the population [3, 35] a correct diagnosis and follow-ups is important in order to evaluate persistence of FHS. This evaluation could be performed within the school health care in early school age [53].

### Strengths and limitations

The strength of our study is that we compared HRQL between children with and without FHS in the same large population-based cohort and by using both generic and disease-specific HRQL questionnaires. By using generic HRQL questionnaire it was possible to compare HRQL among children with FHS and without FHS [54]. Because the classification of children into different FHS phenotypes was performed after completion of the HRQL questionnaire, the children’s responses were not affected by receiving a new diagnosis. Another strength is that the children answered the HRQL questionnaire themselves, since disagreement between child and parent-proxy have been reported [55]. A limitation is that the disease specific questionnaire FAQLQ-TF is primarily developed for IgE-mediated food allergy [13] and does not cover other subgroups of FHS. The Swedish FAQLQ-TF version has not yet been validated but has been used in a previous Swedish study [28]. Regardless, FAQLQ-TF was used in our study because, to our knowledge, there is no available questionnaire in Swedish that is designed to describe adolescents’ own perspective of living with FHS. Despite the current study was based on a large population-based cohort with extremely high participation rate, we were lacking power for some analyses. Regarding the disease-specific HRQL, we found trends with those having current food allergy reporting the lowest HRQL although the differences did not reach statistical significance. Furthermore, the sample size did not allow analyses of HRQL in relation to number of foods avoided.

## Conclusions

We hypothesised that total avoidance of milk, egg, fish or wheat due to food hypersensitivity would have a negative impact on HRQL in children, though this could not be verified. Instead, HRQL was similar in 12–13 year-old children with and without FHS in this population-based study. Poor HRQL was most common in the current food allergy phenotype in the domain Emotional impact and the total FAQLQ. Our study emphasizes the importance of identifying children with current food allergy, the FHS phenotype having impaired HRQL.

## Additional files

**Additional file 1.** Characteristic of participants and non-participants with food hypersensitivity.

**Additional file 2.** Characteristic of participants and non-participants without food hypersensitivity.

**Additional file 3.** Mean and median scores in FAQLQ-TF domains among children with food hypersensitivity and by sex.

## Abbreviations

FAQLQ-TF: Food Allergy Quality of Life Questionnaire Teenager Form
FHS: food hypersensitivity
HRQL: Health Related Quality of Life
IgE: immunoglobulin E
IQ: interquartile range
KIDSCREEN-52: Health-Related Quality of Life Screening instrument for children and adolescents
MID: clinical minimal important difference
SPT: skin prick testing
