# Supplementary material for: Health Related Quality of Life among schoolchildren aged 12–13 years in relation to food hypersensitivity phenotypes: a population-based study
Source: Clin Transl Allergy. 2017 Jul 3;7:20. doi: 10.1186/s13601-017-0156-9 (PMC5494861; doi:10.1186/s13601-017-0156-9)
Supplement: Supplementary file 1 — Additional file 1. Characteristic of participants and non-participants with food hypersensitivity. [file 13601_2017_156_MOESM1_ESM.docx]

|  |  |  |  |  |  |
| --- | --- | --- | --- | --- | --- |
|  |  |  |  |  |  |
|  | **Additional file 1.** |  |  |  |  |
|  | Characteristic of participants and non-participants with food hypersensitivity. | | | |  |
|  |  |  |  |  |  |
|  |  | Participants | Non participants | p-value |  |
|  |  | (n=75) | (n=50) |  |  |
|  |  | % (n) | % (n) |  |  |
|  | Girls | 58.7 (44) | 50.0 (25) | 0.340 |  |
|  | Asthma | 25.3 (19) | 22.0 (11) | 0.669 |  |
|  | Rhinitis | 28.4 (21) | 26.0 (13) | 0.771 |  |
|  | Eczema | 32.0 (24) | 30.0 (15) | 0.813 |  |
|  | Heridity asthma | 40.0 (30) | 32.0 (16) | 0.364 |  |
|  | Heredity rhinitis | 50.7 (38) | 48.0 (24) | 0.770 |  |
|  | Heredity eczema | 36.0 (27) | 22.0 (11) | 0.096 |  |
|  | Heredity FHS | 50.7 (38) | 36.0 (18) | 0.106 |  |
|  | Any positive SPT* | 46.3 (25) | 46.7 (14) | 0.974 |  |
|  | **Living conditions** |  |  |  |  |
|  | Current living: House | 86.1 (62) | 81.3 (39) |  |  |
|  | Apartment | 13.9 (10) | 18.8 (9) | 0.068 |  |
|  | Single parent household | 8.0 (6) | 10.0 (5) | 0.699 |  |
|  | Father smoke | 13.5 (10) | 20.8 (10) | 0.286 |  |
|  | Mother smoke | 10.8 (8) | 14.9 (7) | 0.507 |  |
|  |  |  |  |  |  |
|  | * Based on the 54 participants and the 30 non participants | | |  |  |
|  | with food hypersensitivity who took part in the skin prick testing. | | |  |  |
|  |  |  |  |  |  |
|  |  |  |  |  |  |
