# Supplementary material for: Health Related Quality of Life among schoolchildren aged 12–13 years in relation to food hypersensitivity phenotypes: a population-based study
Source: Clin Transl Allergy. 2017 Jul 3;7:20. doi: 10.1186/s13601-017-0156-9 (PMC5494861; doi:10.1186/s13601-017-0156-9)
Supplement: Supplementary file 2 — Additional file 2. Characteristic of participants and non-participants without food hypersensitivity. [file 13601_2017_156_MOESM2_ESM.docx]

|  |  |  |  |  |  |  |
| --- | --- | --- | --- | --- | --- | --- |
|  | **Additional file 2.** | |  |  |  |  |
|  | Characteristic of participants and non-participants with unrestricted diet. | | | | | |
|  |  |  |  |  |  |  |
|  |  |  | Participants | Non-participants | p-value |  |
|  |  |  | (n=209) | (n=111) |  |  |
|  |  |  | (%) n | (%) n |  |  |
|  | Girls |  | 48.3 (101) | 43.2 (48) | 0.386 |  |
|  | Asthma |  | 6.2 (13) | 8.4 (9) | 0.525 |  |
|  | Rhinitis |  | 6.2 (13) | 5.5 (6) | 0.766 |  |
|  | Eczema |  | 10.5 (22) | 7.3 (8) | 0.332 |  |
|  | Heridity asthma | | 21.1 (44) | 20.7 (23) | 0.945 |  |
|  | Heridity rhinits | | 42.6 (89) | 34.2 (38) | 0.146 |  |
|  | Heredity eczema | | 21.5 (45) | 20.7 (23) | 0.866 |  |
|  | Heredity FHS | | 21.1 (44) | 12.6 (14) | 0.062 |  |
|  | Any positive SPT* | | 33.0 (69) | 36.0 (40) | 0.587 |  |
|  | **Living conditions** | |  |  |  |  |
|  | Current living: House | | 79.6 (156) | 71.7 (76) |  |  |
|  | Apartment | | 20.4 (40) | 28.3 (30) | 0.174 |  |
|  | Single parent household | | 10.2 (21) | 16.7 (18) | 0.099 |  |
|  | Father smoke | | 13.7 (28) | 15.7 (16) | 0.645 |  |
|  | Mother smoke | | 18.2 (37) | 15.2 (16) | 0.510 |  |
|  |  |  |  |  |  |  |
|  | * All participants and non-participants without food hypersensitivity | | | | |  |
|  | took part in the skin prick testing. | | |  |  |  |
|  |  |  |  |  |  |  |
