# Supplementary material for: Health Related Quality of Life among schoolchildren aged 12–13 years in relation to food hypersensitivity phenotypes: a population-based study
Source: Clin Transl Allergy. 2017 Jul 3;7:20. doi: 10.1186/s13601-017-0156-9 (PMC5494861; doi:10.1186/s13601-017-0156-9)
Supplement: Supplementary file 3 — Additional file 3. Mean and median scores in FAQLQ-TF domains among children with food hypersensitivity and by sex. [file 13601_2017_156_MOESM3_ESM.docx]

|  | **Additional file 3.** |  |  |  |  |  |  |
| --- | --- | --- | --- | --- | --- | --- | --- |
|  | Mean and median scores in FAQLQ-TF domains among children with food hypersensitivity by sex. | | | | | |  |
|  |  | |  |  |  |  |  |
|  |  |  |  |  |  |  |  |
|  |  | All (n=74) | Girls (n=43) | Boys (n=31) |  |  |  |
|  |  | Mean/Median | Mean/Median | Mean/Median | p-value* | p-value** |  |
|  | Allergen Avoidance | 3.67/3.63 | 3.75/3.66 | 3.57/3.60 | 0.579 | 0.622 |  |
|  | and Dietary restrictions |  |  |  |  |  |  |
|  | Risk of Accidental | 2.86/2.73 | 2.90/2.66 | 2.78/2.83 | 0.711 | 0.996 |  |
|  | Exposure |  |  |  |  |  |  |
|  | Emotional | 3.84/3.71 | 3.97/3.71 | 3.66/3.57 | 0.324 | 0.427 |  |
|  | Impact |  |  |  |  |  |  |
|  | Total | 3.51/3.39 | 3.60/3.45 | 3.40/3.43 | 0.496 | 0.734 |  |
|  | FAQLQ score |  |  |  |  |  |  |
|  |  |  |  |  |  |  |  |
|  | * statistical significant differences in sex was measured by independent-sample T-test | | | | | |  |
|  | ** statistical significant differences in sex was measured by Mann Whitney U test | | | | | |  |
|  |  |  |  |  |  |  |  |
|  |  |  |  |  |  |  |  |
